# Significance of the E3 ubiquitin protein UBR5 as an oncogene and a prognostic biomarker in colorectal cancer

**DOI:** 10.18632/oncotarget.22531

**Published:** 2017-11-20

**Authors:** Zhongdong Xie, Han Liang, Jinmeng Wang, Xiaowen Xu, Yan Zhu, Aizhen Guo, Xian Shen, Fuao Cao, Wenjun Chang

**Affiliations:** ^1^ Department of Environmental Hygiene, Second Military Medical University, Shanghai, China; ^2^ Department of General Surgery, First Affiliated Hospital, Wenzhou Medical University, Zhejiang, China; ^3^ Department of Dermatology, Wenzhou Central Hospital, Zhejiang, China; ^4^ Department of Colorectal Surgery, Changhai Hospital, Second Military Medical University, Shanghai, China; ^5^ Department of Pathology, Changhai Hospital, Second Military Medical University, Shanghai, China; ^6^ Department of General Practice, Yangpu Hospital, Tongji University School of Medicine, Shanghai, China

**Keywords:** colorectal cancer, UBR5, immunohistochemistry, prognostic biomarker, ubiquitin-proteasome system

## Abstract

The E3 ubiquitin protein UBR5 has been implicated in the regulation of multiple biological functions and has recently emerged as a key regulator of the ubiquitin-proteasome system (UPS) in cancer. However, the clinical significance and biological function of UBR5 in colorectal cancer (CRC) are poorly understood. In this study, we compared the expression pattern of UBR5 between CRC and adjacent normal tissues and found that UBR5 expression was frequently elevated in CRC, possibly through chromosomal gains. Using three CRC patient cohorts, we found that patients with high UBR5 mRNA levels, UBR5 gene amplification, or high nuclear UBR5 protein levels had poor prognoses. Multivariate analysis showed that the alterations in UBR5 were independent predictors of CRC prognosis with the TNM stage as a confounding factor. Furthermore, knockdown of UBR5 prevented the proliferation, colony formation, migration, and invasion of CRC cells in cell culture models. An *in vivo* animal model further confirmed that UBR5 knockdown reduced the growth of CRC tumors. In conclusion, our study is the first to systematically investigate the clinical and biological significance of UBR5 and to conclude that an elevated UBR5 level plays an oncogenic role and may be a potential prognostic marker in CRC.

## INTRODUCTION

Colorectal cancer (CRC) is one of the most common and lethal malignancies, with approximately one million new cases diagnosed annually worldwide [1]. Among these CRC cases, approximately 14-25% are accompanied by distant metastasis [2], which usually results in a poor prognosis. Surgery remains the mainstay of curative treatment. However, a subset of patients will develop local recurrences and metachronous metastases after resection of the primary tumor [3], which is the main cause of CRC-associated death. It is well known that prognoses and chemotherapy responses are quite heterogeneous among the CRC patients [4], and an effective criterion is urgently needed for the stratification of patients. Among the large number of biomarkers that have been investigated in previous studies, microsatellite instability (MSI) is the only consistent marker for the prediction of CRC prognosis [5, 6]. At present, pathological staging remains the most reliable method for the prognostic stratification of CRC patients and the selection of neo-adjuvant treatments [4, 6], but this method is imperfect and its prognostic value has been recently challenged [7]. Therefore, more effective biomarkers are needed for people to predict CRC prognosis and to perform treatment stratification.

The ubiquitin-proteasome system (UPS) is a critical regulator of proteostasis and cell signaling, which, when disrupted, can result in abnormal protein levels, protein-protein interactions, and protein localization. The UPS has been associated with many diseases, including cancer [8, 9]. Among the three known components of the UPS, E3 ubiquitin ligases are largely responsible for determining substrate specificity and ubiquitin chain topology. Targeting E3 ligases for cancer therapy is attracting substantial interest [10–12]. In CRC, many E3 ligases, including APC, RNF43, and FBW7, have been reported to act as tumor suppressors. Inactivating mutations in the genes encoding APC and RNF43 are frequently observed in sporadic CRC. Although mutations of the 2 genes are mutually exclusive, they have similar functions in suppressing WNT signaling [13–15]. Inactivating mutations in the gene encoding FBW7 can cause chromosome instability and drug resistance [16–18], and the gene is also associated with a poor prognosis. Moreover, other E3 ligases, such as Mule, RNF111, CHIP, NEDD4L, and NRDP1, are also abnormally expressed in CRC [19, 20] and have potential functions as tumor suppressors. In contrast, some E3 ligases also exhibit oncogenic activities in CRC. Alterations in SKP2 and UHRF2 are strongly associated with aggressive phenotypes and indicate a poor prognosis in CRC patients [19, 20]. Additionally, FBXL20 and beta-TrCP1 are positively correlated with the activity of the WNT signaling pathway [21, 22]. Therefore, although complex, the functions of the E3 ligase family in CRC are very important, and this family should be closely investigated as a potential promising class of biomarkers in CRC.

The E3 ubiquitin protein UBR5 (EDD1), which is a nuclear phosphoprotein [23, 24], has been implicated in regulation of the DNA damage response, β-catenin activity, metabolism, transcription, and apoptosis [25]. This protein has recently emerged as a key regulator of the UPS in cancer [25]. Chromosomal amplification involving the gene encoding UBR5 is a common alteration in many cancer types [25–27] and occurs mostly in the form of allelic imbalance, resulting in increased UBR5 mRNA expression. Additionally, elevated UBR5 expression has been shown to mediate metastasis of triple-negative breast cancer (TNBC) [27] and cisplatin resistance [28, 29]. Ovarian cancer patients with high UBR5 expression usually have a poor prognosis [30]. Therefore, UBR5 likely plays an oncogenic role in the cancer types mentioned above. In contrast, inactivating mutations in the UBR5 gene are observed in approximately 18% of mantle cell lymphoma cases [31]. Thus, UBR5 is a key regulator of cell signaling that has been strongly implicated in cancer, although whether UBR5 promotes or inhibits tumor progression is somewhat unclear [26, 28, 29, 32–34]. Clues from studies on the function of UBR5 in CRC have been controversial [32, 33, 35]. Therefore, extensive work on this topic is essential. In this study, we investigated the clinical significance of UBR5 with 3 CRC cohorts and clearly demonstrated that UBR5 expression is frequently elevated in CRC and that patients with high UBR5 levels in tumors have a worse prognosis than patients with low UBR5 levels in tumors. Our data show that UBR5 may be a promising prognostic biomarker for CRC. Furthermore, with a series of experiments, we found that knockdown of UBR5 not only significantly suppressed the growth, migration, and invasion of CRC cells *in vitro* but also significantly reduced the tumor weights in a xenograft model. Our study is the first to systematically explore the function of UBR5 in CRC and provide substantial evidence to support the role of UBR5 as an oncogene in CRC.

## RESULTS

### Elevated UBR5 mRNA expression in CRC

**Figure 1:**
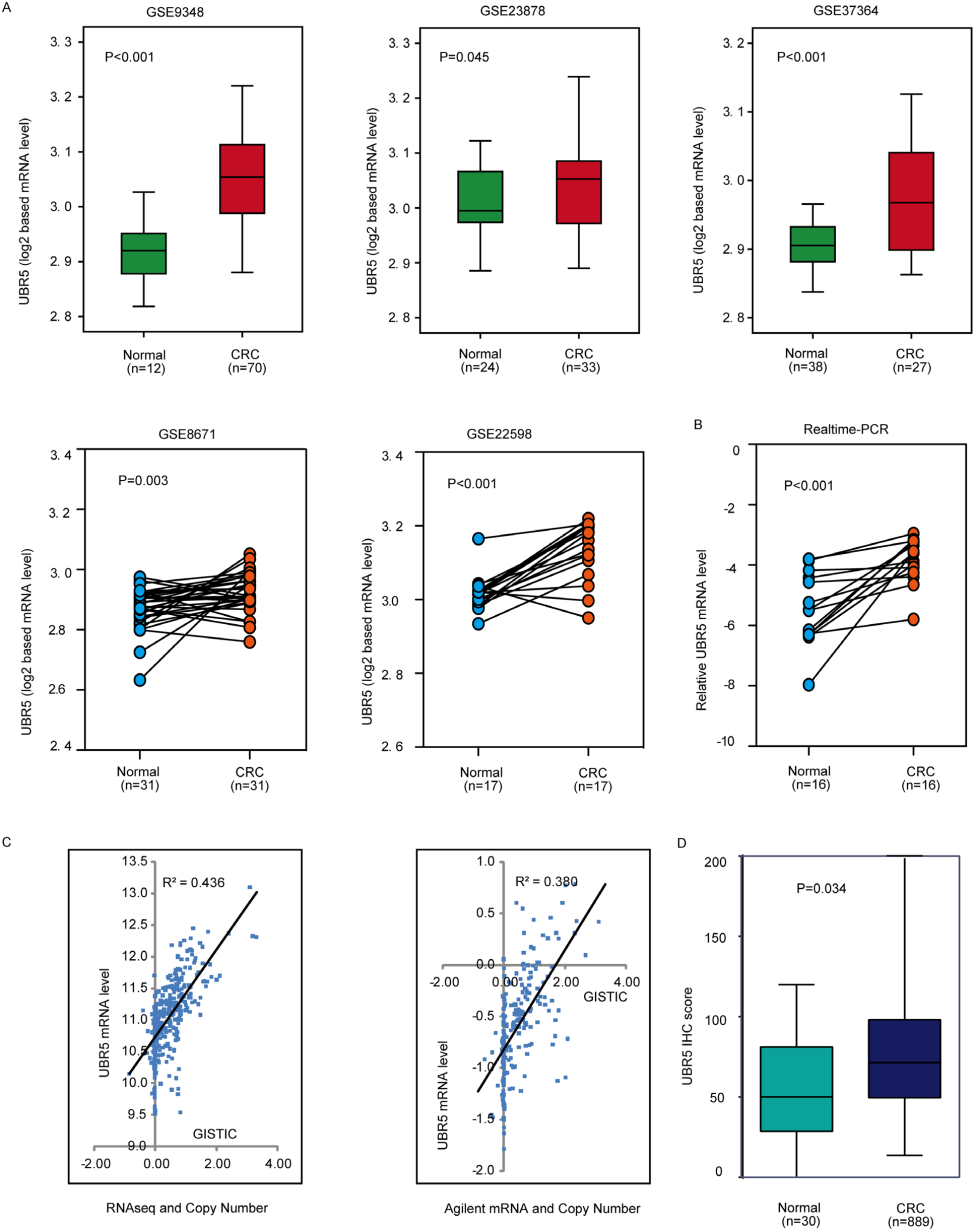
The UBR5 protein and mRNA expression patterns and the linear link between mRNA expression and copy numbers were explored **(A)** Using 5 data sets, the differences in the UBR5 expression levels were explored between the CRC and normal tissues. CRC, colorectal cancer. **(B)** UBR5 transcripts were measured by quantitative real-time PCR in the tumor and normal specimens for 16 CRC samples. **(C)** The link between mRNA expression and the copy number of UBR5 was analyzed. **(D)** UBR5 protein expression was measured in different tissues by semi-quantitative immunohistochemistry based on the UBR5 IHC score. All *P* values are presented in the figure.

To explore the expression pattern of UBR5 in CRC, we first analyzed five publicly available microarray data sets with cancer and normal tissue specimens. Interestingly, the UBR5 mRNA levels were consistently significantly up-regulated in the cancer tissues compared to the levels in the adjacent normal tissues (all *P* values < 0.05) in all data sets (Figure 1A). We validated the UBR5 mRNA expression pattern in the cancer tissues using qPCR (Figure 1B). Because changes in mRNA expression could be caused by somatic copy number variations (CNVs), we investigated the association between the UBR5 mRNA levels and the corresponding copy numbers of the gene. According to the UBR5 copy numbers obtained from the GISTIC2 threshold, gains and losses of chromosomal regions harboring the UBR5 gene were observed in 58% and 2.1% of CRC cases in The Cancer Genome Atlas (TCGA) cohort, respectively, indicating that the DNA copy of UBR5 was significantly amplified (*P* < 0.001) in CRC. In the gene-dosage analysis, significant positive correlations were found between the UBR5 gene copies and the UBR5 mRNA levels measured by RNA-seq (n = 376, R^2^ = 0.436, *P* < 0.001) or Agilent array (n = 217, R^2^ = 0.380, *P* < 0.001), which indicated that copy number gains might be an important contributor to the elevated UBR5 expression in CRC (Figure 1C).

### High UBR5 mRNA expression in tumors predicts poor survival

**Figure 2:**
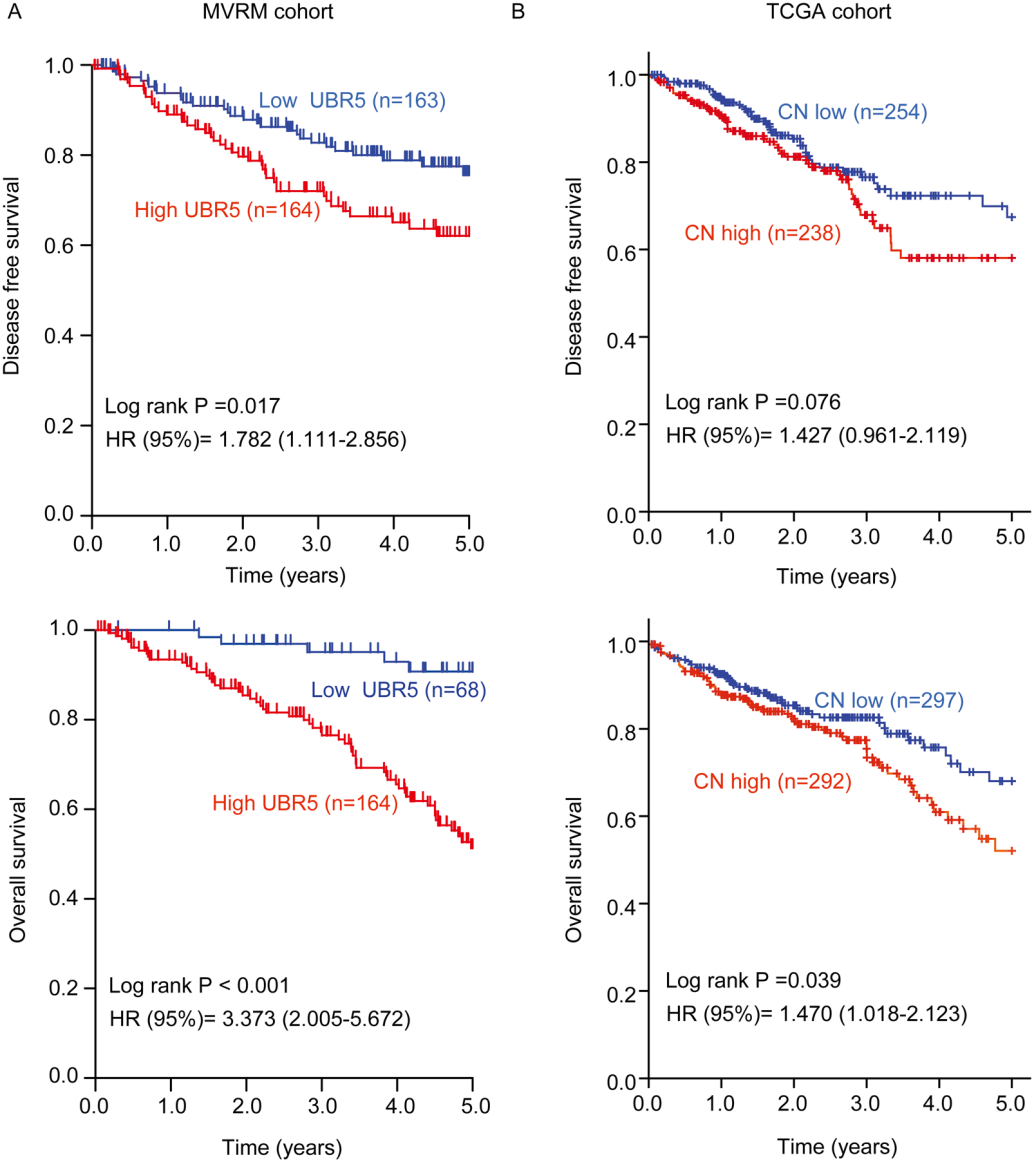
High UBR5 mRNA expression or a high UBR5 copy number in tumors predicts poor survival in patients with CRC **(A)** In the MVRM cohort, the patients were dichotomized into subgroups with high or low UBR5 mRNA expression according to the median UBR5 expression level across the cohort. **(B)** In TCGA CRC cohort, patients were dichotomized into subgroups with high or low UBR5 copy numbers according to the median UBR5 copy number across the cohort. Disease-free survival (DFS) and overall survival (OS) are presented. Log-rank *P* values and HRs from the univariate Cox analysis are shown. CN, copy number.

We further explored the association between UBR5 mRNA expression and the survival outcomes of CRC patients in the Moffit-Vanderbilt-Royal Melbourne (MVRM) [36] and TCGA cohorts. Based on the median UBR5 mRNA expression level in the 2 cohorts, we divided the patients into two subgroups with high or low tumor UBR5 expression levels. In the MVRM cohort, Kaplan-Meier analysis showed that patients with high UBR5 mRNA levels in tumors had shorter disease-free survival (DFS) and overall survival (OS) than patients with low UBR5 mRNA levels in tumors (Figure 2A). Additionally, compared with patients with low UBR5 mRNA levels, patients with high UBR5 mRNA levels tended to have an advanced TNM stage (*P* = 0.003). Multivariate Cox analysis showed that a high UBR5 mRNA level in tumors is an independent predictor of DFS, with a hazard ratio (HR) of 1.925 (95% confidence interval (CI), 1.278-2.899), and of OS, with an HR of 5.321 (95% CI, 2.210-13.35), with age, sex, and the TNM stage included as the confounding variables (Supplementary Table 3). However, we could not determine a similar association using UBR5 mRNA expression data measured by RNA-seq or Agilent array in TCGA cohort (data not shown). Interestingly, the associations observed in the MVRM cohort regarding the effect of UBR5 on survival outcomes were validated in TCGA cohort using univariate analysis when the UBR5 copy number (CN) data were used. However, the multivariate analysis did not identify a high UBR5 copy number as an independent variable in TCGA cohort (Supplementary Table 3). As shown in the Kaplan-Meier plot (Figure 2B), patients with high UBR5 copy numbers had shorter OS (*P* = 0.039) than patients with low UBR5 copy numbers. Considering the gene-dosage relationship between UBR5 mRNA expression and copy numbers (Figure 1C) together with the association between the UBR5 copy number and the survival outcomes, we concluded that patients with high UBR5 mRNA levels in tumors had a poor prognosis.

### High nuclear localization of the UBR5 protein in tumors predicts poor survival

**Figure 3:**
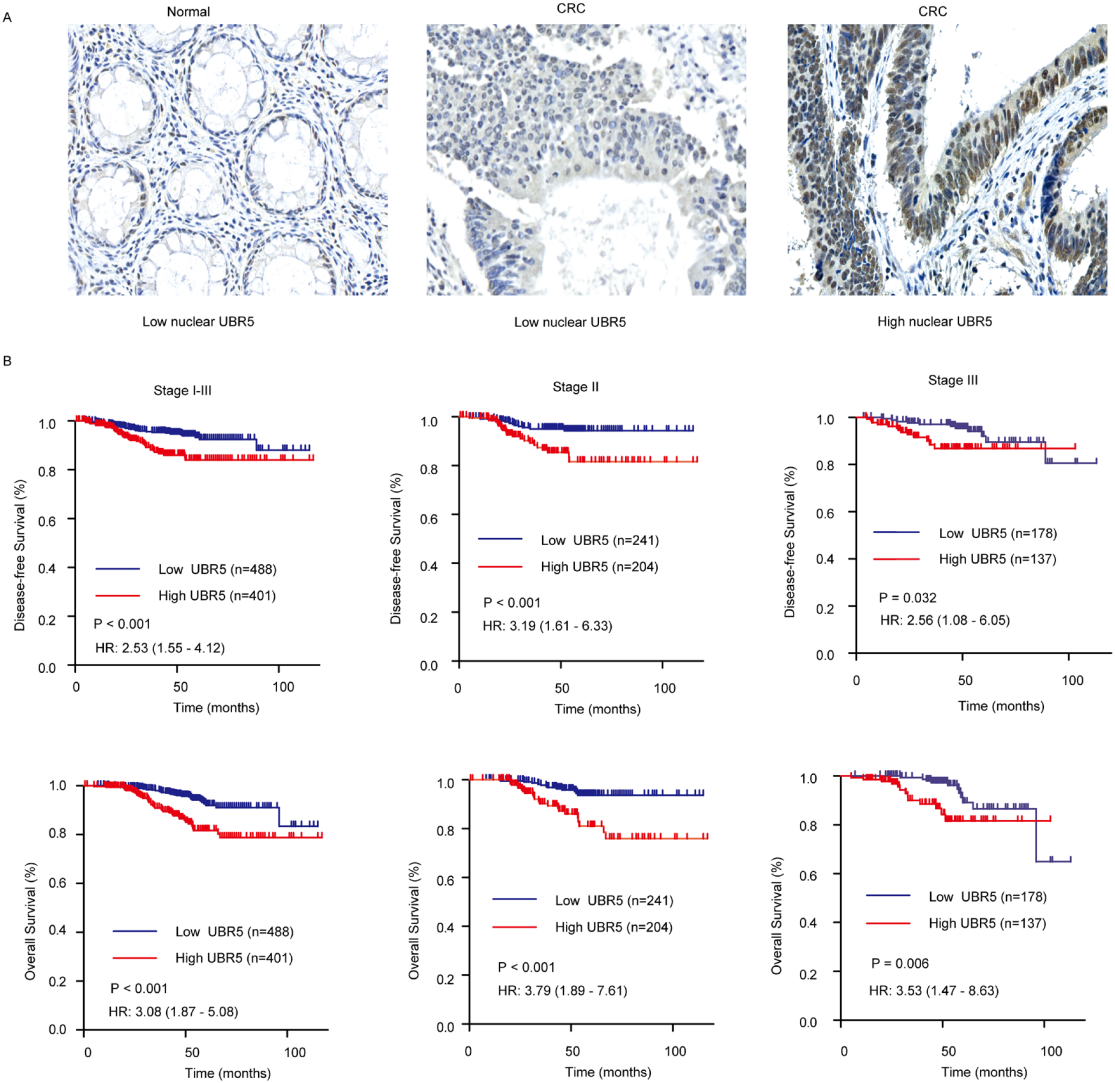
Association between nuclear UBR5 expression and patient survival in CRC **(A)** Representative images for UBR5 immunostaining in colorectal tissues. Bar, 50 μm. **(B)** A high IHC score for nuclear UBR5 expression predicts poor survival in patients with CRC. Patients in the Changhai cohort with stage I-III, stage II, or stage III tumors were dichotomized into subgroups with high or low nuclear UBR5 protein expression according to the IHC score cut-off value for nuclear UBR5 expression. DFS and OS are presented. Log-rank *P* values and HRs from the univariate Cox analysis are shown.

Compared with the UBR5 mRNA, UBR5 protein expression is mainly localized to the nucleoplasm [24] [23]and may be more closely associated with the biological function of UBR5. We found that UBR5 mRNA expression in 7 CRC cell lines was significantly correlated to their corresponding total (r = 0.723, *P* < 0.001) and nuclear protein levels (r = 0.899, *P* < 0.001), as shown in Supplementary Figure 1. Thus, we investigated the association between UBR5 protein expression and patient survival using immunohistochemistry (IHC) on our tissue microarrays (TMAs). As shown in Figure 3A, UBR5 immunostaining was mainly distributed in the cytoplasm and nuclei of colorectal epithelial cells. The cytoplasmic staining of UBR5 was not different between the cancer and adjacent normal tissues and was also not associated with patient survival (data not shown). In contrast, nuclear UBR5 protein expression was significantly elevated in CRC compared to the expression in the adjacent normal tissues (*P* = 0.034) (Figure 1D). The nuclear staining of UBR5 in CRC tissues was also associated with patient survival. Kaplan-Meier analysis showed that patients with high nuclear UBR5 protein expression levels had shorter DFS and OS than patients with low nuclear UBR5 protein expression levels (all *P* values < 0.05) (Figure 3B). High nuclear UBR5 protein expression was also associated with high serum CA19-9 levels (Table 1). Cox multivariate analysis showed that a high nuclear UBR5 protein expression level was an independent predictor of DFS, with an HR of 2.244 (95% CI, 1.348-3.736), and of OS, with an HR of 2.549 (95% CI, 1.532-4.243), with the grade, TNM stage, and age included as the confounding variables. The details are provided in Table 2. For patients with stage I CRC, we did not find any association between nuclear UBR5 expression and patient survival. However, we consistently found that high nuclear UBR5 protein expression was indicative of a poor prognosis in patients with stage II or stage III CRC. The data are presented in Figure 3B.

**Table 1:** Characteristics of patient with CRC dichotomized by nuclear UBR5 protein in Changhai cohort

Characteristics	UBR5-low (n=488)	UBR5-high (n=401)	P-value^*^
Age (years), mean (SD)	60.95 (12.67)	61.26 (12.35)	0.714^†^
Sex (n(%))			
women	200 (41.0)	166 (41.4)	0.901
men	288 (59.0)	235 (58.6)	
Disease location (n(%))			
Colon	255 (52.3)	228 (56.9)	0.170
Rectum	233 (47.7)	173 (43.1)	
Differentiation grade (n(%))			
Well	7 (1.4)	15 (3.7)	0.080^‡^
Moderately	414 (84.8)	336 (83.8)	
Poorly	62 (12.7)	42 (10.5)	
Missing	5 (1.1)	8 (2.0)	
Number of lymph nodes resected at surgery (n (%))			
<12	396 (81.1)	321 (80.0)	0.680
≥12	92 (18.2)	80 (20.0)	
TNM stage (n(%))			
I	69 (14.1)	60 (14.9)	0.479^‡^
II	241 (49.4)	204 (50.9)	
III	178 (36.5)	137 (34.2)	
Adjuvant chemotherapy (n(%))			
Yes	384 (78.7)	295 (73.6)	0.545
No	79 (16.2)	54 (13.5)	
Missing	25 (5.1)	52 (12.9)	
Serum CEA (ng/mL), median (range)	3.66 (0-9398.0)	3.46 (0-205.5)	0.757^‡^
Serum CA19-9 (U/mL), median (range)	13.50 (0-1000.0)	11.14 (0-1200.0)	0.005^‡^

^*^χ^2^ test or Fisher's exact test.

^†^ Student t test.

^‡^ Mann-Whitney U test (non-parametric). Missing values are excluded for all statistic tests.

CA19-9, carbohydrate antigen 19-9; CEA, carcinoembryonic antigen; TNM, tumour-node-metastasis

**Table 2:** Cox regression analysis of nuclear UBR5 protein expression and clinicopathological covariates in changhai cohort

Characteristics	Disease-free Survival				Overall Survival			
	Univariate		Multivariate		Univariate		Multivariate	
	HR (95%CI)	p Value	HR (95%CI)	P Value	HR (95%CI)	P Value	HR (95%CI)	P Value
UBR5-high *vs.* UBR5-low	2.429 (1.497-3.942)	<0.001	2.244(1.348-3.736)	0.002	2.835 (1.748-4.598)	<0.001	2.549 (1.532-4.243)	<0.001
Age (≥60 *vs.* <60)	1.216 (0.750-1.973)	0.428	1.016 (0.601-1.721)	0.951	1.193 (0.736-1.935)	0.474	1.079 (0.637-1.828)	0.779
Sex (male *vs.* female)	1.518 (0.915-2.518)	0.106	1.513 (0.883-2.593)	0.132	1.588 (0.957-2.635)	0.074	1.632 (0.944-2.70)	0.080
Location (colon *vs.* rectal)	1.335 (0.831-2.144)	0.232	1.242 (0.734-2.102)	0.420	1.242 (0.773-1.994)	0.371	1.145 (0.673-1.949)	0.617
TNM, per increase in stage	1.003 (0.706-1.426)	0.985	1.202 (0.785-1.842)	0.397	1.016 (0.713-1.448)	0.928	1.208 (0.786-1.855)	0.388
Grade, per increase in stage	2.233 (1.282-3.889)	0.005	2.988 (1.556-5.737)	0.001	2.054 (1.180-3.578)	0.011	2.841 (1.480-5.452)	0.002
Chemo (yes *vs.* no)	0.573 (0.324-1.013)	0.055	0.538 (0.282-1.027)	0.060	0.576 (0.325-1.024)	0.060	0.517 (0.270-0.989)	0.046
Resected lymph node (≥12 *vs.* <12)	1.032 (0.553-1.927)	0.922	1.582 (0.765-3.279)	0.216	1.010 (0.540-1.890)	0.975	1.435 (0.696-2.950)	0.329
Serum CEA(ng/ml) (≥5 *vs.* <5)	1.650 (1.025-2.654)	0.039	1.282 (0.743-2.213)	0.372	1.671 (1.038-2.689)	0.034	1.338 (0.776-2.309)	0.295
Serum CA199(U/ml) (≥37 *vs.* <37)	1.724 (0.972-3.060)	0.063	1.805 (0.925-3.522)	0.083	1.729 (0.974-3.070)	0.062	1.750 (0.900-3.402)	0.099

HR, hazard ratio; CI, confidence interval.

### UBR5 promotes the growth and aggressiveness of CRC cells

**Figure 4:**
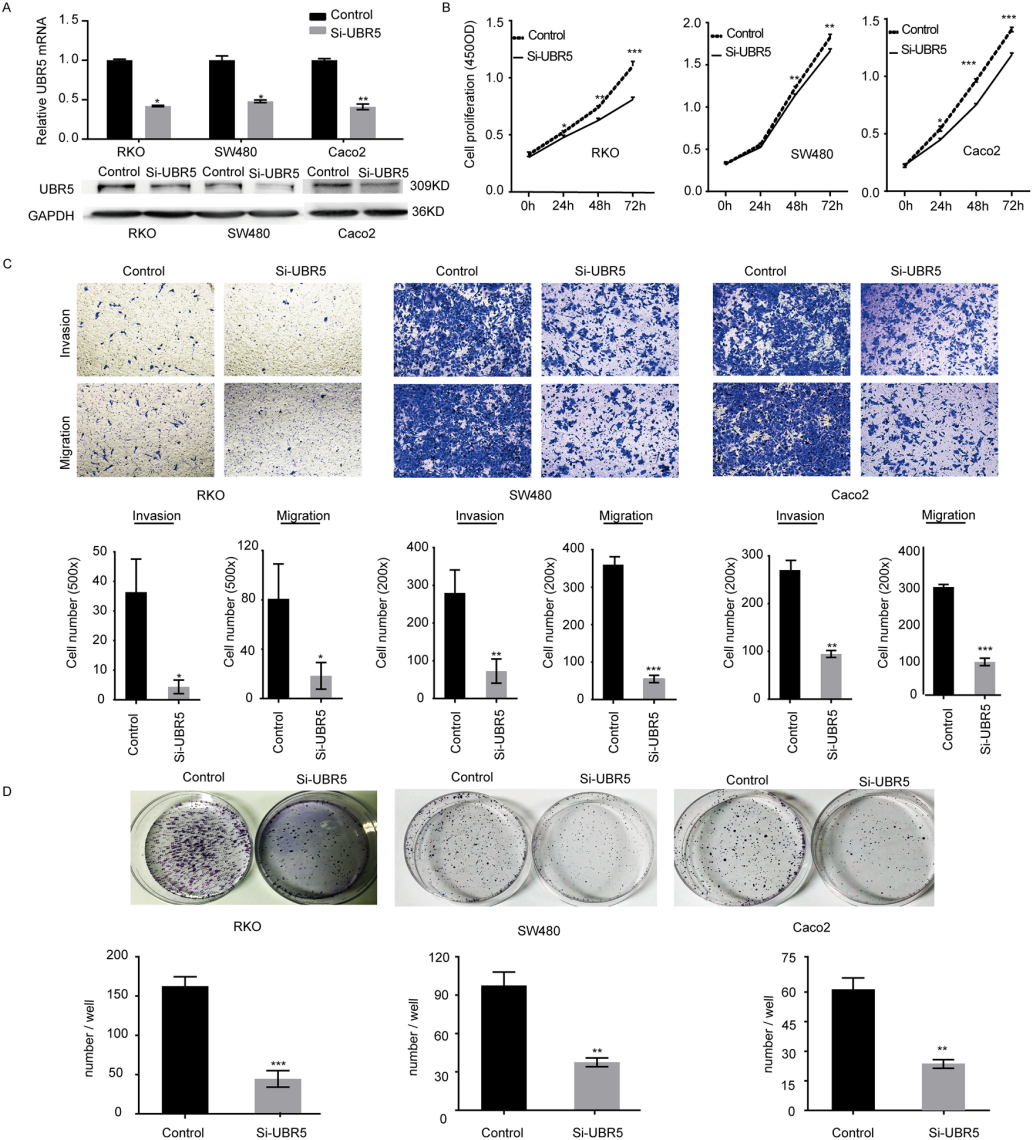
UBR5 promotes the growth and aggressiveness of CRC cells **(A)** The efficiencies of the selected UBR5 siRNA pairs in reducing UBR5 expression in the indicated cells were examined by qPCR (upper panel) and western blotting (lower panel). **(B-D)** The cell proliferation (B), migration and invasion (C), and colony formation (D) of CRCs were examined. (^*^P < 0.05; ^**^P < 0.01; ^***^P < 0.001)The error bars represent the standard error of the mean obtained from three independent experiments. The statistical analysis was performed using one-way analysis of variance and Mann–Whitney U test appropriately.

To explore the biological roles of UBR5 in CRC progression, we assessed the effects of UBR5 knockdown on the proliferative potential and aggressiveness of CRC cells. UBR5 is highly expressed in SW480, RKO, Caco2, and DLD-1 cells but is expressed at relatively low levels in HCT116, COLO205, and LoVo cells (Supplementary Figure 1). Therefore, we knocked down UBR5 expression in SW480, RKO, and Caco2 cells using siRNAs and/or shRNAs. We synthesized four pairs of siRNAs and shRNAs targeting UBR5 and selected the most effective siRNA duplexes and shRNAs for subsequent use (Figure 4A and Supplementary Figure 2A). Then, we assessed the proliferation, colony formation, migration, and invasion abilities of CRC cells after UBR5 knockdown. Compared with the control cells, UBR5 knockdown via shRNA expression and siRNA transfection resulted in significantly lower proliferation, colony formation, migration, and invasion abilities in the SW480, RKO, and Caco2 cell lines (Figure 4B-4D and Supplementary Figure 2B-2D). To exclude the possibility of off-target effects, we transiently transfected GFP-UBR5 expression plasmids into SW480/UBR5 shRNA and RKO/UBR5 shRNA cells after stimulated in the absence of doxycycline and found that UBR5 over-expression rescues the effects of UBR5 knockdown on cell proliferation (Supplementary Figure 2E). Therefore, UBR5 promotes the growth and aggressiveness of CRC cells *in vitro*.

### Knockdown of UBR5 reduces CRC growth in an animal model

**Figure 5:**
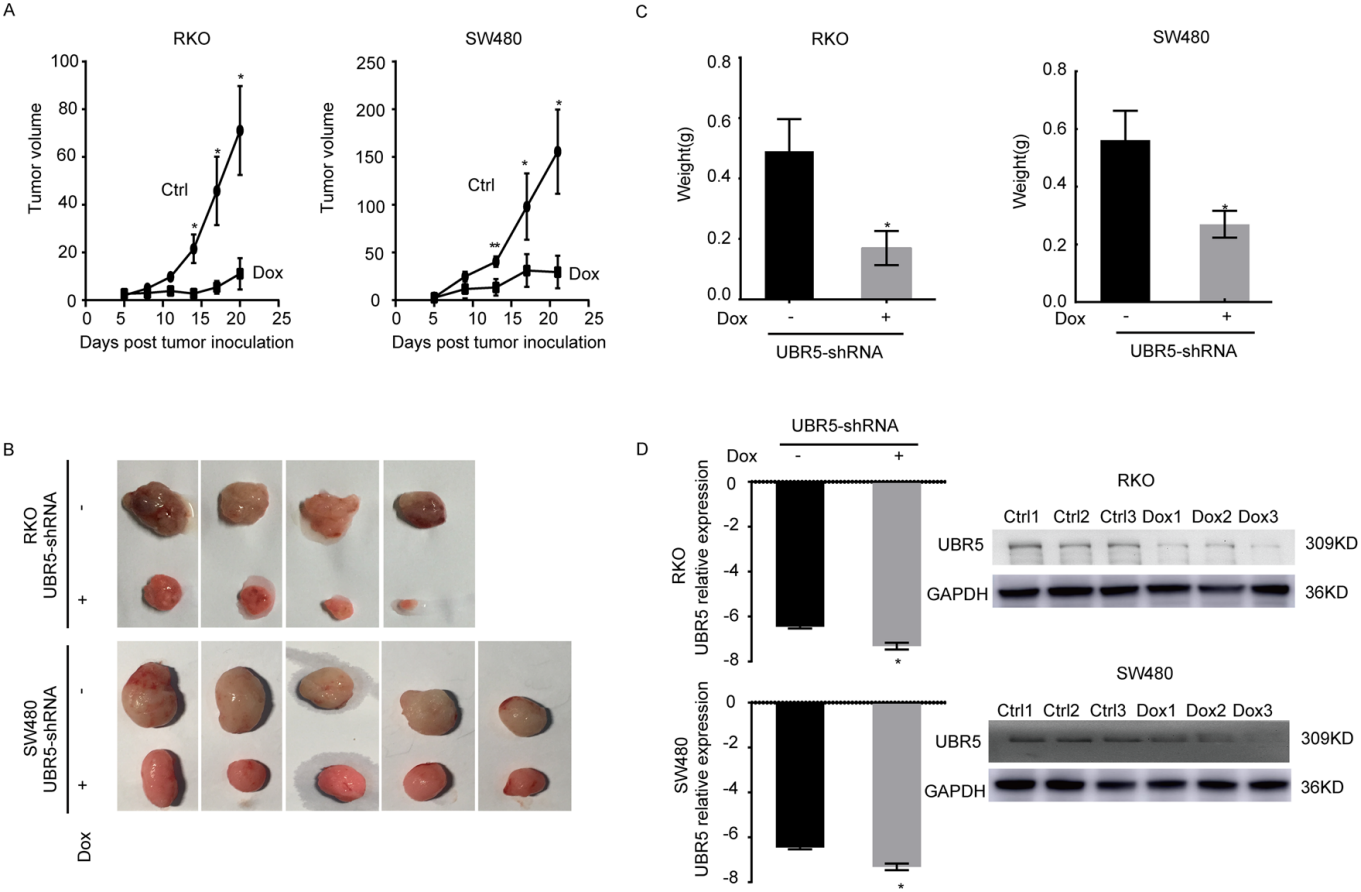
Knockdown of UBR5 reduces the growth of CRC xenografts in BALB/c nude mice The effect of UBR5 knockdown in SW480 and RKO cells on the xenograft model was assessed by evaluating the tumor volume **(A, B)** and weight **(C)** in a xenograft model. **(D)** The UBR5 expression levels were examined by qPCR and western blotting in the xenograft tumors with or without doxycycline-inducible UBR5 shRNA expression. The ctrl group was treated in the absence of doxycycline (Dox-), and the Dox group was supplied doxycycline (Dox+) in the drinking water. As lanes show, the Control group included the ctrl1, ctrl2, and ctrl3 xenograft tumor specimens, and the Dox group included the Dox1, Dox2, and Dox3 specimens. Ctrl, Control. All statistical tests were two-sided. ^*^P < 0.05, ^**^P < 0.01, ^***^P < 0.001. The error bars represent the standard error of the mean obtained from three independent experiments. The statistical analysis was performed using one-way analysis of variance and Mann-Whitney U test appropriately.

To assess whether UBR5 knockdown reduces tumorigenicity *in vivo*, we subcutaneously injected SW480/UBR5 shRNA cells or RKO/UBR5 shRNA cells into BALB/c nude mice with doxycycline (Dox), without doxycycline (Control) in the drinking water. During the first 8 days post-injection, no significant difference in tumor size was observed between the Dox and control groups. However, the mice injected with the UBR5-knockdown cells appear to slow tumor growth around 14 days post-injection than control group. Both tumor size (Figure 5A) and weight (Figure 5C) were significantly lower for UBR5 shRNA-expressing cells than those of control animals at 20-21 days after injection, and reduced UBR5 expression was maintained in the tumor tissues until the end of the experiment (Figure 5D). Therefore, low UBR5 expression reduces tumor growth *in vivo*, which is consistent with our *in vitro* and clinical findings.

## DISCUSSION

UBR5 is an important E3 ubiquitin ligase that is attracting the attention of investigators in relation to multiple cancer types [25–31]; however, few studies have investigated UBR5 in CRC [32, 33, 35]. The clinical significance and biological function of UBR5 in CRC are unknown. In this study, we explored the expression pattern of UBR5 in CRC using publicly available microarray expression profiles. All five data sets examined consistently showed that the UBR5 mRNA level was higher in CRC than in adjacent normal tissues. Using qPCR, we further verified the expression pattern of UBR5 in fresh cancer and adjacent normal tissue samples. Considering that the elevation in the UBR5 mRNA level in CRC might be caused by chromosomal aberrations, we assessed the association between the UBR5 mRNA expression levels and the somatic copy numbers of UBR5 in a CRC cohort from TCGA. As previously reported in breast and ovarian cancer [25, 26], the chromosomal region encoding UBR5 is frequently amplified in CRC, and this finding was observed in approximately 50% of the cases in TCGA cohort. Furthermore, the UBR5 mRNA levels and copy numbers were positively correlated in TCGA CRC cohort, which indicated that elevated UBR5 mRNA expression in CRC might result from gains in the chromosome region encoding UBR5. Because genes with somatic mutations or chromosomal aberrations are likely to be cancer drivers [34, 37], we proposed that UBR5 might serve as a key molecule in the initiation and/or progression of CRC.

Recent studies in breast and ovarian cancer have clearly established associations between UBR5 expression and the prognosis of or metastasis in patients [27–30]; however, similar study has not been performed in CRC. Next, we carefully assessed 2 publicly available cohorts with survival data (the MVRM and TCGA cohorts) and the Changhai cohort to investigate the association between UBR5 alterations and patient survival. Using the median UBR5 mRNA expression level as the cut-off point, the patients from the MVRM and TCGA cohorts were divided into high and low UBR5 expression subgroups. In the MVRM cohort, patients with high UBR5 mRNA expression levels in tumors had shorter DFS and OS than the patients with low UBR5 mRNA expression levels. However, a similar association was not observed using the gene expression data from TCGA cohort. Interestingly, patients with a gain in the UBR5 chromosomal region usually had shorter DFS and OS than patients with a loss of or unchanged UBR5 chromosomal region. Therefore, due to the existence of a gene-dosage relationship between UBR5 mRNA expression and chromosomal variations, high UBR5 mRNA levels in tumors were associated with a poor prognosis. The inconsistencies regarding the UBR5 transcriptional data in the prediction of the CRC prognosis between the MVRM and TCGA cohorts might be attributed to the differences in the methods used to assess mRNA expression and unpredictable discrepancies in the follow-up of patients between the 2 cohorts.

Through examination of 7 CRC cell lines, we found a significant correlation between the UBR5 mRNA and total protein expression levels and between UBR5 mRNA expression and nuclear UBR5 protein expression. The result suggested that the UBR5 protein might be valuable as a prognostic mark in CRC. To explore the association, we assessed the expression levels and subcellular localization of the UBR5 protein using IHC on TMAs from the Changhai cohort. The UBR5 protein was mainly distributed in the cytoplasm and nuclei of CRC epithelial cells. Given that the biological function of a protein is determined not only by its expression level but also by its cellular location, we investigated the association between the cytoplasmic or nuclear UBR5 protein levels and patient survival. The cytoplasmic UBR5 protein levels were not associated with the survival of CRC patients; however, nuclear UBR5 protein expression was significantly associated with patient survival. Patients with high nuclear UBR5 protein levels had shorter DFS and OS than patients with low nuclear UBR5 protein levels. Our results are consistent with those of previous studies [26, 30, 34, 37–39], indicating that the nuclear UBR5 protein has a more important function than the cytoplasmic UBR5 protein. Thus, our results from the 3 cohorts almost consistently demonstrated that UBR5 expression in CRC is indicative of poor survival. Additionally, the CRC prognosis is dependent on the tumor stage and grade to some extent. After normalizing for the TNM stage, a high UBR5 mRNA level in the MVRM cohort and a high nuclear UBR5 protein level in changhai cohort consistently predicted a poor prognosis in the multivariate analyses.

A previous study demonstrated that UBR5 could serve as a tumor suppressor by increasing the stability of APC [32]. However, knockdown of UBR5 significantly promoted the growth and aggressiveness of CRC in our *in vitro* and *in vivo* models. Recently, UBR5 has been reported to directly interact with β-catenin or its subunits and activate WNT signaling [35]. The frequency of inactivating mutations in APC in CRC is greater than 80%, and thus, the increase in the expression of abnormal APC by UBR5 may be unnecessary. Together with previous evidence from breast and ovarian cancer [26, 27, 29, 37], the results of our experiments and those from the CRC cohorts strongly suggest that UBR5 likely functions as an oncogene in CRC. However, the underlying mechanism by which UBR5 regulates CRC tumorigenicity warrants further investigation.

Our study has some limitations. First, the reason for the inconsistencies in the prognostic role of UBR5 mRNA between the MVRM and TCGA cohorts was hard to explain. Second, some important prognostic factors, such as MSI and extramural venous invasion, were not included in our analyses, resulting in an incomplete inclusion of variants in the multivariate Cox model.

In summary, we systematically investigated the potential role of UBR5 and provided the first evidence for the clinical and biological significance of UBR5 in CRC. UBR5 may be used as a potential predictive biomarker for risk stratification of localized CRC. Furthermore, we provided evidence to support the oncogenic role of UBR5 in CRC tumorigenesis. However, the mechanism underlying the oncogenic role of UBR5 in CRC remains unclear, and further work is needed. We conclude that UBR5 may be a promising prognostic biomarker with potential for use as a therapeutic target in CRC.

## MATERIALS AND METHODS

### Genomic data mining

Raw data from 7 data sets (GSE8671, GSE9348, GSE22598, GSE23878, GSE37364, GSE14333, and GSE17538) were downloaded from the Gene Expression Ominous (GEO) database (https://www.ncbi.nlm.nih.gov/geo/). Each data set was examined using the Affymetrix plus 2.0 platform (Santa Clara, CA, USA), and the corresponding gene expression profiles were extracted with the fRMA package [40] in the R 3.2.0 environment. Among the data sets, 5 (GSE8671, GSE9348, GSE22598, GSE23878, and GSE37364) were used to investigate the differential expression of UBR5 between cancer and normal tissues. Two data sets (GSE14333 and GSE17538) that were annotated with survival information and some clinical information, including the TNM stage and chemotherapy, were combined into one data set (MVRM cohort) for the survival analysis, and the duplicated samples were manually removed. The gene-dosage relationship between UBR5 expression and copy numbers was explored using processed data from TCGA cohort with 736 patients. The prognostic importance of the UBR5 mRNA expression levels and copy numbers was also investigated in TCGA cohort. The corresponding genomic data and clinical information, including survival information, were downloaded from UCSC Xena (http://xena.ucsc.edu/). Among the 736 patients in TCGA CRC cohort, 616 had CNV data, 434 had mRNA expression data obtained using RNA-seq, and 246 had mRNA expression data obtained using Agilent arrays. The characteristics of the MVRM and TCGA cohorts are presented in Supplementary Table 1.

### Patient characteristics

Sixteen fresh paired cancer and adjacent normal tissues were obtained from patients with CRC after curative surgery and stored at -80°C for mRNA extraction. Commercial TMAs containing formalin-fixed, paraffin-embedded (FFPE) specimens from 889 stages I-III CRC patients (Outdo Biotech, Shanghai, China) were used for the IHC analysis. Patients received curative surgery in Changhai Hospital, Second Military Medical University (Shanghai, China) between January 2007 and October 2012. The baseline characteristics of these patients, including age, sex, disease location, TNM staging at surgery, and rule-based postoperative chemotherapy (FOLFOX regimen), are documented in Table 1. TNM staging was performed according to the American Joint Committee on Cancer staging manual (seventh edition). Fewer than 5% of the patients with rectal cancer in the cohort received preoperative radiotherapy. DFS and OS were assessed at 6-month intervals by 2 investigators. All participants are self-reported Han Chinese. This study was approved by the Institutional Review Boards of Changhai Hospital. Written informed consent was obtained from each patient.

### qPCR, western blotting, and immunohistochemistry

Total RNA was extracted from cells or tissues using the TRIzol reagent (Takara, Shiga, Japan) according to the manufacturer's protocol. Complementary DNA (cDNA) was synthesized with the PrimeScript™ RT Master Mix Reverse Transcription kit (RR036A, Takara, Dalian, China) and then subjected to qPCR with the SYBR Green PCR Master Mix (RR820A, Takara) on the Roche LightCycler 480 II machine (Roche, Indiana, USA). The mRNA level of each gene was calculated using Ct values and normalized to corresponding GAPDH mRNA level for each sample. The primer sequences and PCR program are given in Supplementary Table 2. Western blotting was performed with rabbit polyclonal antibodies to human UBR5 (1:2000, ab70311, Abcam) and human GAPDH (1:1000, AP0063, Bioworld Technology, St. Louis Park, MN, USA) as previously described. Antibodies against human UBR5 at a final concentration of 1:1000 were used for IHC in the TMAs [27]. The immunostaining protocol was based on the manufacturer's recommendations. Antigens were retrieved with citrate buffer (pH 6.0). UBR5 protein expression was semi-quantitated using the H-score method as previously reported [36]. The intensity of the cytoplasmic and nuclear staining of UBR5 (0, 1 +, 2 +, and 3 +) and the total percentage of positive epithelial cells were independently scored by 2 authors in a blinded manner. A minimum of 100 cells were evaluated to calculate the IHC score. The equation IHC score = (%_1_ × 1) + (%_2_ × 2) + (%_3_ × 3) was used to calculate the IHC score for each specimen. The average IHC score from the two observers was applied for the analysis. The association between UBR5 expression and the survival outcomes was analyzed by an investigator who did not participate in the scoring process.

### Cell culture and transfection

The human CRC cell lines (DLD-1, Caco2, HCT116, RKO, SW480, LoVo, and COLO205) were obtained from American Type Culture Collection (ATCC, Manassas, VA, USA). All cells were maintained in Dulbecco's modified Eagle's medium (Gibco, Grand Island, NY, USA) supplemented with 10% heat-inactivated fetal bovine serum (Gibco), 100 U/mL of penicillin, and 100 mg/mL of streptomycin in a 5% CO_2_ incubator at 37°C. Negative control (NC) or UBR5-targeting siRNA (siUBR5) duplexes were designed and synthesized by GenePharma (GenePharma, Co., Ltd., Shanghai, China); the siRNA sequences are listed in Supplementary Table 2. The CRC cells were transfected with siUBR5 or the scrambled siRNA at a final concentration of 20 nM/mL using the Lipofectamine® RNAiMAX reagent (Invitrogen, Carlsbad, CA, USA) according to the manufacturer's protocol. Transient over-expression of UBR5 in CRC cells was achieved using the GFP-UBR5 plasmid (52050, Addgene, Cambridge, MA USA) and Lipofectamine 2000 (Invitrogen) according to the manufacturer's protocol.

### Construction of stable CRC cells with inducible UBR5 knockdown

Based on the sequences of shRNAs specific to UBR5, different microRNA (miR)-30-mediated shRNAs targeting UBR5 were generated by adding a miR-30 loop and appropriate flanking sequences as previously described; the shRNAs were synthesized as single-stranded DNA templates [41]. Then, the templates were subjected to PCR amplification with primers containing Xho1 or BamH1 restriction sites as shown in Supplementary Table 2. The PCR products were purified and cloned into the doxycycline-inducible lentiviral vector Pinducer10 [38] between the Xho1 and BamH1 sites and referred to as Pinducer10-shRNA/UBR5. The new vectors were confirmed by sequencing. The lentiviral vector Pinducer10-shRNA/UBR5 was co-transfected into 293T cells with Lenti-X HTX Packaging mix plasmids using the Lenti-X™ HTX Packaging System (Clontech Laboratories, Inc., CA, USA). Then, supernatant containing lentiviral particles was harvested, filtered, and directly used to infect CRC cells in the presence of 8 μg/mL of polybrene. Stable cell lines were obtained after selection with 1 μg/mL of puromycin for approximately 2 weeks. The expression of UBR5 shRNA was induced by the addition of 1 μg/mL of doxycycline (dox), and knockdown of UBR5 was verified by real-time PCR and western blotting.

### Cell proliferation, colony formation, migration, and invasion assays

For the proliferation assay, CRC cells were seeded into 96-well plates at a density of 3000 cells per well for 24 h, and then, were transfected with UBR5 siRNAs and NC siRNAs or dox was added. The number of viable cells at 24 h, 48 h, and 72 h after transfection or dox addition was assayed using Cell Counting Kit-8 (Dojindo, Kumamoto, Japan). The absorbance at 450 nm was measured as an indicator of the cell population. For colony formation, siRNA transfection or dox induction was performed in 6-well plates at a cell density of 1.0 × 10^3^/well. The resulting colonies were fixed with ice-cold methanol and stained with crystal violet solution for counting after approximately 2 weeks of culture. To determine the invasion and migration capacity of CRC cells, the transwell assay was carried out. Boyden chambers with matrigel bedding (BD Pharmingen, San Jose, CA, USA) were used to determine cell invasion, and chambers without matrigel bedding were used to determine cell migration. Tumor cells (2×10^5^) in serum-free medium were placed in the upper champers and 800μL of complete medium was added to the lower chambers. After 16h incubation for migration assay and 24h incubation for invasion assay, cells that had invaded or migrated into the lowers surface of the membrane were fixed with 4% paraformaldehyde and stained with 0.5% crystal violet. Photographs of five randomly selected fields were captured, and the cells were counted. The experiments were independently conducted three times.

### *In vivo* tumor growth

Five-week-old male BALB/c nude mice were purchased from the Laboratory Animal Center of Shanghai at the Academy of Science (Shanghai, China). The mice were acclimated in a pathogen-free facility (12-h light and dark cycles) for one week prior to the injection of CRC cells. For the establishment of human tumors in the BALB/c nude mice, stable inducible UBR5 shRNA-RKO or -SW480 cells (5 × 10^6^ cells / mouse) were subcutaneously injected into the proximal midline of the dorsa in 10 mice respectively. Then, the mice injected with UBR5 shRNA-RKO or -SW480 were randomly divided into the test and control groups. The mice were provided free access to drinking water with or without 3 mg/mL of doxycycline throughout the experiment. The tumor sizes were measured every 3-5 days, and the tumor volumes were calculated as the length × width × height × 0.52. The efficiency of UBR5 silencing was examined by western blotting of tissue lysates after tumor excision. The animal protocol was approved by the Institutional Animal Care and Use Committee of the Second Military University.

### Statistical analysis

To compare UBR5 expression between the CRC and adjacent normal tissues, we used independent sample t-tests for non-paired samples and paired t-tests for paired samples. Categorical data, such as sex and the tumor differentiation grades were compared and analyzed using the χ^2^ test or Mann-Whitney U test. For the survival analysis, patient subgroups divided according to the UBR5 copy number or mRNA or protein expression levels were compared using the Kaplan-Meier method and univariate and multivariate Cox proportional hazards models. The log-rank test was used to assess the statistical significance of the Kaplan-Meier curves. All statistical tests were two-sided and were performed with the R software version 3.2.0 and SPSS version 16.0.2 for Windows (SPSS, Chicago, IL, USA). Statistical significance was set at *P* < 0.05.

## SUPPLEMENTARY MATERIALS FIGURES AND TABLES


