# Supplementary material for: Significance of the E3 ubiquitin protein UBR5 as an oncogene and a prognostic biomarker in colorectal cancer
Source: Oncotarget. 2017 Nov 20;8(64):108079–92. doi: 10.18632/oncotarget.22531 (PMC5746127; doi:10.18632/oncotarget.22531)
Supplement: Supplementary file 1 [file oncotarget-08-108079-s001.pdf]

Significance of the E3 ubiquitin protein UBR5 as an oncogene and a prognostic biomarker in colorectal cancer

SUPPLEMENTARY MATERIALS

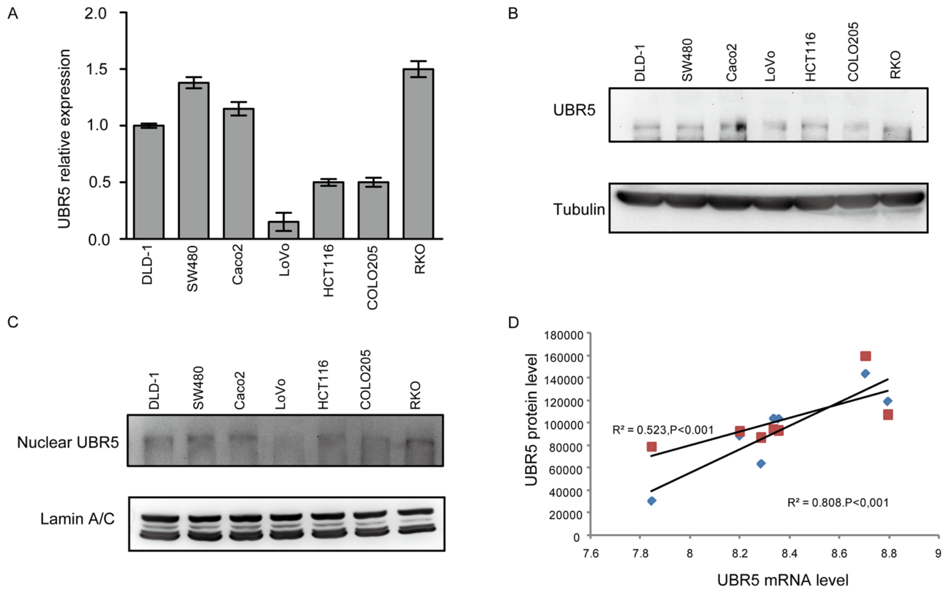

Supplementary Figure 1: Significant correlation was existed between the mRNA and protein expression of UBR5 in 7 CRC cells. **(A)** Baseline UBR5 expression in the CRC cell lines examined by qPCR. **(B-C)** Western blot was performed to determine UBR5 protein level. Tubulin or lamin A/C was used as loading control. **(D)** The link between UBR5 mRNA and total UBR5 protein level or nuclear protein level was explored. Red dots exhibited the link between UBR5 mRNA and total UBR5 protein level, and blue dots represented the association between UBR5 mRNA and nuclear protein level. *P* value was presented in the figure.

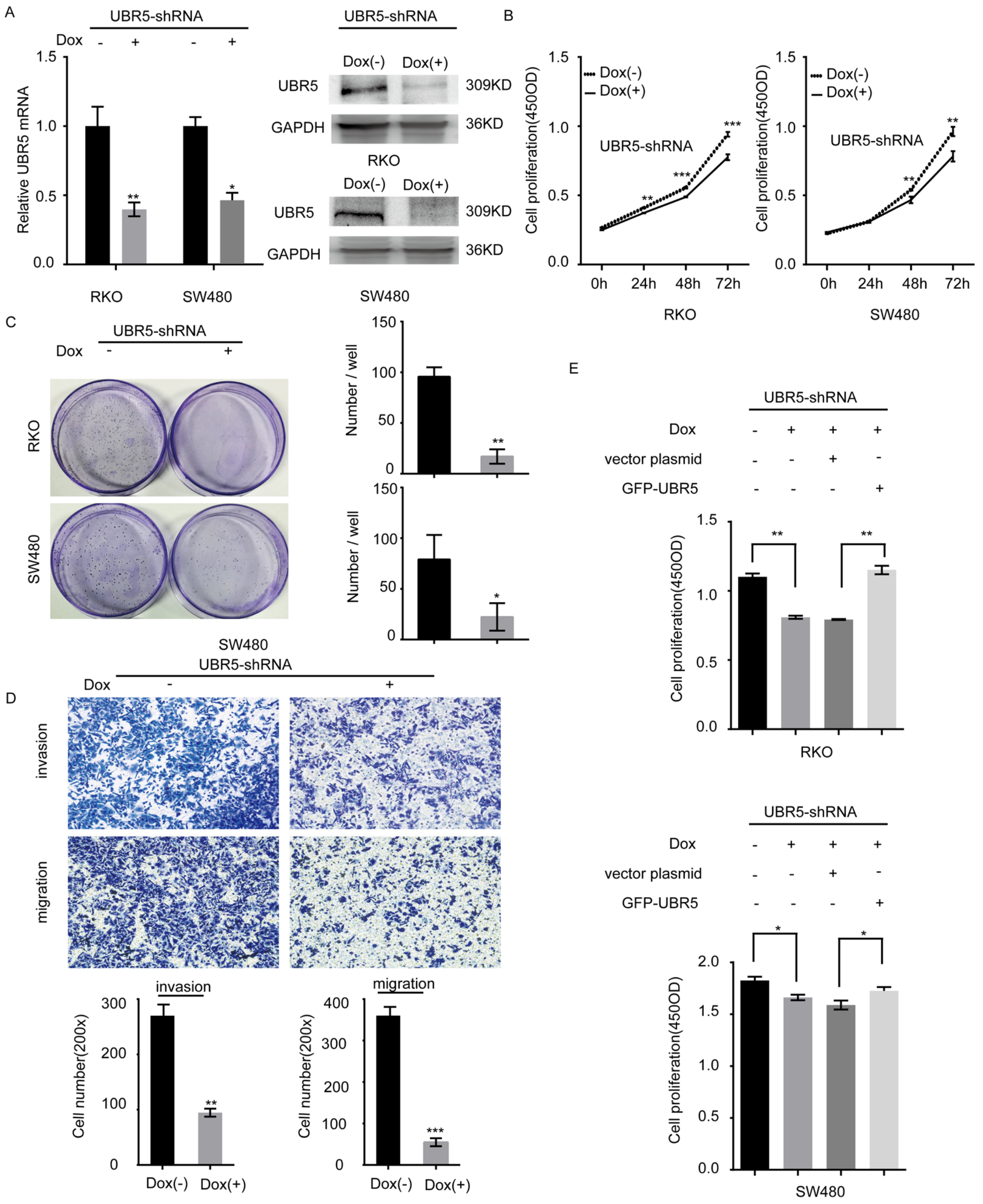

Supplementary Figure 2: Doxycycline-inducible knockdown of UBR5 inhibits the growth and aggressiveness *in vitro*. **(A)** Western blot and qPCR showing UBR5 knockdown in SW480, RKO cells. Colon cancer cells were transfected with lentivirus expressing shRNA targeting UBR5 with or without induction of doxycycline (Dox) for assay. **(B)** Proliferation assay for uninduced control and induced UBR5 knockdown colon cancer cells. **(C)** Cell colony formation assay for uninduced control and induced UBR5 knockdown colon cancer cells. **(D)** Transwell migration and invasion assay for uninduced control and induced UBR5 knockdown colon cancer cells. The error bars represent the standard error of mean obtained from three independent experiments. **(E)** UBR5 overexpression rescued the impaired proliferation of the indicated CRC cells after stimulated with doxycycline after 24h. Statistical analysis was performed using one-way analysis of variance and Mann-Whitney U tests appropriately. All statistical tests were two-sided. $^{*}p<0.05$, $^{**}p<0.01$, $^{***}p<0.001$.

Supplementary Table 1: Characteristics of patient with CRC dichotomized by mRNA expression or chromosomal aberration of UBR5

Characteristics	MVRM cohort (n=327)			TCGA cohort (n=616)		
	UBR5-low (n=163)	UBR5-high (n=164)	P-value	Copy Number-low (n=309)	Copy Number-high (n=307)	P-value
Age (years), mean (SD)	66.37 (12.14)	64.36 (18.86)	0.164†	67.23 (12.95)	65.26 (12.59)	0.057†
Sex (n(%))						
women	89 (54.6)	73 (44.5)	0.068*	153 (49.7)	136 (44.6)	0.381*
men	74 (45.4)	91 (55.5)		155 (50.3)	169 (55.4)	
TNM stage (n(%))						
I	24 (14.7)	22 (13.4)	0.003‡	58 (18.8)	47 (15.3)	0.002‡
II	70 (42.9)	44 (26.8)		125 (40.5)	98 (31.9)	
III	50 (30.7)	61 (37.2)		83 (26.9)	95 (30.9)	
IV	19 (11.7)	37 (22.6)		32 (10.4)	57 (18.6)	
NA	0 (0.0)	0 (0.0)		11(3.6)	10 (3.3)	

*χ^2 test or Fisher's exact test.

† Student t test.

‡ Mann-Whitney U test (non-parametric). Missing values are excluded for all statistic tests.

TNM, tumour-node-metastasis

Supplementary Table 2: Sequences of siRNA and shRNA and and qPCR primers for this study

	**siRNA duplexs :5' to 3'**	**Concentration used**	**Work or not**
UBR5-siRNA1	GCAGUGUUCCUGCCUUCU (sense)<br>AGAAGGCAGGAACACUGC (antisense)	20nM	Yes
UBR5-siRNA2	GCGACUCUCCAUGGUUUCU(sense)<br>AGAAACCAUGGAGAGUCGC(antisense)	20nM	no
Control siRNA	UUCUCCGAACGUGUCACGU(sense)<br>ACGUGACACGUUCGGAGAA(antisense)	20nM	/
	**Primers: 5'-3'**	**PCR program**	
UBR5	AGGACGAGAAGGAAAGCACC(forward)<br>TCAGAAACTTCTCGTAACCTGTCA(reverse)	Denatured 2min at 95°C, and followed by 40 cycles of 95°C for 10s, 60°C for 34s and 72°C for 10s	
GAPDH	GGAGCGAGATCCCTCCAAAAT(forward)<br>GGCTGTTGTCATACTTCTCATGG(reverse)		
	**shRNA :5' to 3'**		Work or not
UBR5-ShRNA1	ATGACAGCAGAACAACATAATT	/	Yes
UBR5-ShRNA2	AGCCATTAGAAAGAACCACAAA		No
97-mer template	5′-TGCTGTTGACAGTGAGCG-**22merSense-**TAGTGAAGCCACAGATGTA**-22merGuide-**TGCCTACTGCCTCGGA-3′		
5′miR30-XhoI	5′-TACAATACTCGAGAAGGTATATTGCTGTTGACAGTGAGCG-3′		
3′miR30-EcoRI	5′-ACTTAGAAGAATTCCGAGGCAGTAGGCA-3′		

Supplementary Table 3: Cox analysis of the mRNA expression or chromosomal variations of UBR5 and clinicopathological covariates in 2 public cohorts

Characteristics	Disease-free Survival				Overall Survival			
	Univariate		Multivariate		Univariate		Multivariate	
	HR (95%CI)	p Value	HR (95%CI)	P Value	HR (95%CI)	P Value	HR (95%CI)	P Value
**MVRM (n=327)**								
UBR5-high *vs.* UBR5-low	2.104 (1.423-3.113)	< 0.001	1.925 (1.278-2.899)	0.002	4.424 (2.573-16.036)	< 0.001	5.321 (2.210-13.35)	< 0.001
Age (>=60 *vs.* <60)	1.134 (0.635-2.024)	0.671	1.137 (0.632-2.047)	0.668	1.618 (0.974-2.688)	0.063	1.332 (0.788-2.252)	0.285
Sex (male *vs.* female)	1.018 (0.700-1.479)	0.925	1.125 (0.760-1.667)	0.556	1.236 (0.746-2.047)	0.411	1.008 (0.596-1.705)	0.976
TNM, per increase in stage	3.343 (2.574-4.342)	< 0.001	3.294 (2.525-4.298)	< 0.001	1.992 (1.482-2.677)	< 0.001	1.806 (1.338-2.439)	< 0.001
**TCGA (n=616)**								
CNV-high *vs.* CNV-low	1.427 (0.961-2.119)	0.078	1.286 (0.855-1.935)	0.227	1.470 (1.018-2.123)	0.400	1.359 (0.925-1.996)	0.118
Age (≥60 *vs.* <60)	1.342 (0.890-2.028)	0.160	1.289 (0.837-1.988)	0.249	1.750 (1.100-2.784)	0.018	2.584 (1.560-4.279)	< 0.001
Sex (male *vs.* female)	1.502 (1.002-2.247)	0.049	1.520 (1.001-2.315)	0.049	1.057 (0.735-1.519)	0.765	1.215 (0.833-1.773)	0.312
TNM, per increase in stage	1.828 (1.454-2.298)	< 0.001	1.758 (1.397-2.211)	< 0.001	2.109 (1.705-2.609)	<0.001	2.203 (1.771-2.741)	< 0.001

HR, hazard ratio; CI, confidence interval.
